# Relationship Between Out-of-School Physical Activity, Physical Growth and the Development of Motor Skills

**DOI:** 10.3390/children12121582

**Published:** 2025-11-21

**Authors:** Lilyan Vega-Ramírez, María Teresa Pascual-Galiano, Juan José Chinchilla, María Alejandra Ávalos-Ramos

**Affiliations:** EDUCAPHYS Research Group, Department of General and Specific Didactics, Faculty of Education, University of Alicante, 03690 Alicante, Spain; mariateresa.pascual@ua.es (M.T.P.-G.); jj.chinchilla@ua.es (J.J.C.); sandra.avalos@ua.es (M.A.Á.-R.)

**Keywords:** motor competence, physical education, postural control, primary education, sedentary lifestyles

## Abstract

**Highlights:**

**What are the main findings?**

• Levels of competence in the turning motor skill increase with age and with the frequency of extracurricular physical activity.
• A higher BMI is associated with slightly lower performance in turning tasks; after adjusting for age and practice, sex differences are substantially reduced.

**What is the implication of the main finding?**

• Integrating specific turning tasks into PE classes and promoting extracurricular physical activity can enhance overall motor performance in schoolchildren.
• Designing inclusive interventions that consider weight status may help reduce motor competence gaps and foster active participation.

**Abstract:**

**Background/Objectives**: Motor development in middle childhood (9–11 years) is a decisive stage for the consolidation of fundamental motor skills. Among these, turning stands out as a complex stabilizing skill that requires postural control, angular momentum regulation, and sensory integration. However, despite its cross-cutting relevance in physical and sports practice, it has not been studied specifically. **Methods**: This cross-sectional, quantitative study analyzed the execution level of the turn in 214 Spanish students aged 9 to 11, considering its relationship with anthropometric variables (height, weight, and BMI), sex, and the frequency of extracurricular physical-sport practice. **Results**: The results showed mostly average-to-low performance in turning ability, with no linear progression with age; boys outperformed girls at ages 9 and 10, while at age 11 the differences narrowed. Regression analysis showed no significant associations (*p* < 0.05) between turning ability and anthropometric variables or frequency of general physical and sports activity. **Conclusions**: These findings reinforce the originality of the study by highlighting a rarely explored skill and underscore the need for specific pedagogical approaches. Educational and extracurricular programs should incorporate varied tasks designed to stimulate sensory integration, body awareness, and motor control, beyond the mere amount of physical practice.

## 1. Introduction

Motor development is the continuous and dynamic process through which human beings acquire, refine, and automate movement patterns as a result of the interaction between biological maturation (somatic and nervous system growth), learning, and environmental conditions [1,2]. This complex process entails the progressive organization of multiple bodily and sensory systems that act in an integrated manner to produce efficient and context-adapted movements [3].

On the one hand, postural control allows the body to maintain stability both at rest and in motion, adjusting the position of body segments anticipatorily and reactively [4], while inter- and intramuscular coordination optimizes the simultaneous or sequential activation of different muscle groups, reducing unnecessary effort and improving movement precision [5]. On the other hand, balance, understood as the ability to maintain or recover body position in the face of internal or external perturbations, is complemented by spatial perception, which enables the body to be oriented and moved effectively in relation to objects, people, and environmental boundaries [6]. Additionally, movement tempo regulates the speed, sequence, and duration of actions to ensure functionality [7].

In the case of turning, it constitutes a particularly complex stabilizing skill, as it simultaneously combines fine postural adjustments, angular momentum control, and precise management of body inertia. Its efficient execution requires children to anticipate the sequence of movements, modulate speed, and harmoniously coordinate the action of different body segments. Moreover, it involves integrated sensory processing, where the vestibular system detects changes in head position and acceleration, vision provides spatial references for orienting rotation, and proprioception conveys information about the relative position of the limbs and muscle tension in each phase of the turn. This integration enhances technical quality of movement, while also contributing to the development of body awareness and improved spatial orientation [8,9].

In the study of motor development during middle childhood, unlike more static skills such as balance, turning allows us to observe how children respond to active body rotation demands, which are influenced by factors such as gender, anthropometric characteristics, and extracurricular sports practice [10]. Furthermore, turning is a motor skill with high functional value, common in recreational and sports activities, making it a good indicator of motor competence in ecological contexts.

Despite its technical relevance and presence across numerous disciplines, turning has received relatively limited attention in the scientific literature on sport and motor performance, particularly when compared with other fundamental skills such as running, jumping, or linear displacements [11,12]. Most research has focused on general aspects of balance, coordination, or rotation in specific contexts such as gymnastics or dance, without systematically addressing turning as a stabilizing motor skill transversal to multiple sports [13,14]. This scarcity of studies hinders the establishment of clear reference models for its teaching, assessment, and optimization, underscoring the need to expand empirical evidence regarding its biomechanical, neuromotor, and perceptual demands [15].

Nevertheless, in practice, the learning and refinement of turning do not occur in isolation; they are conditioned by the child’s prior experience with other motor skills and by the practice opportunities provided by their environment. Contexts such as school physical education, extracurricular activities, and free play offer varied scenarios for experimenting with turns under different conditions, thereby enriching the motor repertoire and strengthening the adaptability of the neuromotor system. In the case of systematic practice, for example, in sports such as gymnastics, dance, or figure skating, turning plays a central role in technical preparation and is a determining factor for performance [16,17].

In this regard, middle childhood, particularly between the ages of 9 and 11, represents a stage of consolidation of motor capacities and transition toward more specialized movement patterns [18,19]. This period is characterized by increased neuromuscular efficiency, relative strength gains, and improvements in intersegmental coordination, all of which support the execution of more complex motor sequences [20]. Significant changes also occur in body composition, such as increases in height, gains in lean mass, and, in some cases, variations in BMI that may condition motor performance. From a maturational standpoint, these years precede the pubertal growth spurt, a stage during which rapid growth can temporarily disrupt coordination and postural control. Thus, studying this age range allows for the observation of motor performance during a period of relative stability and high motor learning potential, before hormonal changes associated with puberty introduce new developmental variables [21]. Selecting turn analysis as the primary stabilizing skill in this study will enable the identification of performance patterns associated with both anthropometric characteristics and motor practice habits, providing evidence that can guide educational interventions and motor development programs tailored to the needs of this developmental stage.

In this context, the aim of this study was to analyze the execution level of turning on the transverse and horizontal axes in children aged 9 to 11, considering its relationship with physical growth parameters (height, weight, and BMI), sex, and participation in extracurricular physical activity, with particular attention to practice frequency.

## 2. Materials and Methods

This study employed a quantitative cross-sectional design with a descriptive and correlational approach.

### 2.1. Sample

The convenience and availability sample was composed of 214 primary school students (101 boys and 113 girls) from a province in Spain, aged between 9 and 11 years (mean age = 10.04 ± 1.305). For participation in this study, informed consent was obtained from both the students’ parents and the head of the educational institution. This research complied with data protection legislation and received approval from the Ethics Committee of the University of Alicante (UA-2023-07-13).

### 2.2. Instruments

For data collection, the turn test from the Scale for the Assessment of Basic Motor Skills by Fernández et al. [22] was used. This test consists of 15 tasks ranging from simple motor activities to more complex ones. These tasks include turns without and with previous movements, combined with jumps in the longitudinal axis from an elevated place, with various receptions and varying the position of the arms. In addition, turning tasks on the horizontal axis are presented with different positions of start and end of the movement. The tasks, age and levels of difficulty are given by the test itself (Table 1). Table 2 shows the scores on the scale used to assess turning competence.

Weight, height, and body mass index (BMI) were measured using an electronic scale and a stadiometer. Body mass index (BMI, kg/m^2^) was calculated as weight divided by height squared. BMI-for-age Z-scores (BAZ) were then derived using the World Health Organization 2007 growth reference for children and adolescents aged 5–19 years, stratified by sex and age in months. To assess sports habits, a questionnaire composed of three multiple-choice questions was used.

### 2.3. Procedure

After obtaining the relevant authorizations, the sports habits questionnaire was administered by physical education teachers in the presence of the researchers. Once the questionnaire was completed, the researchers measured the weight and height of the participants during the physical education sessions. These measurements were taken individually, with children wearing shorts, a T-shirt, and no shoes. After a week of anthropometric measurements, the motor skill of turning was assessed using the Scale for the Assessment of Basic Motor Skills. This process was carried out by two of the researchers in this study who had previously familiarized themselves with the criteria established in the test following this protocol:

1. Informing students about the evaluations to be carried out.
2. Describing each task of the motor skill assessment scale (turning) to ensure participants understood them.
3. Presenting the tasks in the order specified by the test.
4. Test administration.

If the task was completed correctly, it was scored with 1; otherwise, it was scored with 0. The task could be repeated if necessary. A second attempt was allowed if the first one failed. If a participant failed two consecutive tasks after a successful one, the test was suspended.

### 2.4. Data Analysis

Statistical analyses were performed using IBM SPSS Statistics 29.0 (IBM, Armonk, NY, USA) for Windows. Descriptive statistics (mean and standard deviation) were calculated to characterize turn performance and anthropometric variables within each age group.

Associations between turn competence and quantitative variables (height, weight, BMI, and frequency of physical activity) were examined through multiple linear regression analysis, reporting unstandardized (B) and standardized (β) coefficients, standard errors, determination coefficient (R^2^), and exact *p*-values.

Sex differences in turn performance and frequency of physical activity were assessed using independent-samples t-tests, with Levene’s test applied to verify homogeneity of variances. Effect sizes (Cohen’s d) were reported together with 95% confidence intervals.

Parametric assumptions were verified: normality of distributions (Kolmogorov–Smirnov test and skewness/kurtosis analysis), homogeneity of variances, multicollinearity (VIF < 10), and independence of residuals (Durbin–Watson). A 95% confidence level was applied in all analyses.

## 3. Results

**Figure 1:**
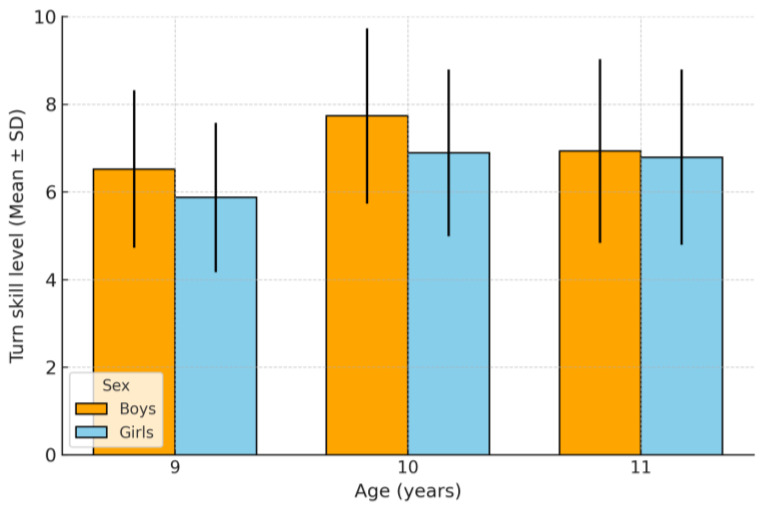
Turn performance by age and sex.

The analysis of turn skill competence level (Table 3 and Figure 1) shows that, on average, students aged 9 to 11 are positioned in a medium-low range, without a linear progression by age. At age 9, the mean was 6.15 points (SD = 1.836), with almost half (47.8%) in the medium-low level and 10.5% in the low level. At age 10, although the mean increased to 7.35 points (SD = 1.836), there was a concerning concentration in the lower levels: 63.8% in medium-low and 23.9% in low, representing the weakest overall profile of the group. At age 11, the mean decreased to 6.85 points (SD = 2.112), but a distribution shift was observed: 41.8% reached the medium level, the percentage in medium-low decreased to 15.5%, although 29.9% remained at the low level.

The dispersion of results was similar across ages, suggesting constant internal variability. Overall, the data indicates that the development of this skill does not follow a continuous improvement pattern with age, and that in some groups, lower levels of performance predominate. This finding may require specific pedagogical interventions to foster progress.

When disaggregating the data by sex, in the 9-year-old group, boys obtained a higher mean turning score than girls (6.52 vs. 5.87), with a greater proportion at the medium-high level (37.9%) and a lower presence at the low level (6.9%). In contrast, girls concentrated on more cases at the medium-low (52.6%) and low levels (13.2%), with less representation in the higher ranges.

At age 10, the mean difference widened in favor of boys (7.73 vs. 6.89). Notably, 18.1% of boys reached a high level compared to only 2.8% of girls. However, in both sexes, the medium-low level was overwhelmingly predominant (63.7% in boys and 63.8% in girls), indicating an overall moderate performance.

At age 11, the means was very similar between boys (6.93) and girls (6.79). In this group, most participants were in the medium level (39.3% in boys and 43.6% in girls), with a lower concentration in the lower levels than in younger ages. Nevertheless, 35.7% of boys and 25.7% of girls remained at the low level, evidencing considerable variability in performance.

Overall, the data show that at ages 9 and 10, boys tend to achieve better results and greater representation in higher levels than girls, while at age 11, sex differences are reduced. A change in the distribution pattern is also observed: from the predominance of the medium-low level at younger ages toward a greater presence in the medium level at older age. This may suggest a general improvement in turn execution, although a significant percentage of students remain in the lower performance levels.

The results show a progressive increase in height and weight in both sexes between the ages of 9 and 11, with a relatively stable BMI (Table 4). Height analysis indicates that boys are slightly taller than girls at age 9, but by age 10 girls reach parity, and at age 11 exact equality is observed. This coincides with the pattern of earlier maturation in girls. Regarding weight, boys weigh more at age 9, but from age 10 onward, girls slightly surpass boys, a trend that persists at age 11. Finally, BMI values remain between 18.8 and 19.9, without marked variation across ages. Girls present slightly higher values in most age groups, which may reflect differences in body composition. Overall, the observed pattern is consistent with prepubertal growth, characterized by continuous development and the advancement of girls in height and weight between ages 10 and 11. Standard deviations indicate greater variability in weight than in height, suggesting individual differences in body mass and possibly in dietary or physical activity habits.

### 3.1. Analysis of Turn Skill Competence Level According to Physical-Sport Activity Habits

Overall, the results indicate that, across all ages, girls tend to participate more in either purely individual or collective physical-sport activities, whereas boys show greater involvement in the combined practice of both types. Significant differences were observed by gender in both the frequency of sports practice and the level of execution of turns (Table 5). Boys reported a higher frequency of physical activity (M = 2.79, SD = 0.97) compared to girls (M = 2.32, SD = 1.05) (*t* (212) = 3.42, *p* = 0.001), with a moderate effect size (d = 0.47). Likewise, boys showed a higher level of turns (M = 7.16, SD = 2.14) than girls (M = 6.51, SD = 1.93) (*t* (212) = 2.32, *p* = 0.021), with a small-moderate effect size (d = 0.30).

### 3.2. Correlational Analysis

Regression models confirmed that turn performance was not significantly predicted by age, anthropometric measures (height, weight, BMI), or frequency of sports practice. The global model explained less than 1% of the variance in turn scores (F (5, 208) = 0.20, *p* = 0.963, R^2^ = 0.005), indicating that these factors had virtually no explanatory power.

**Figure 2:**
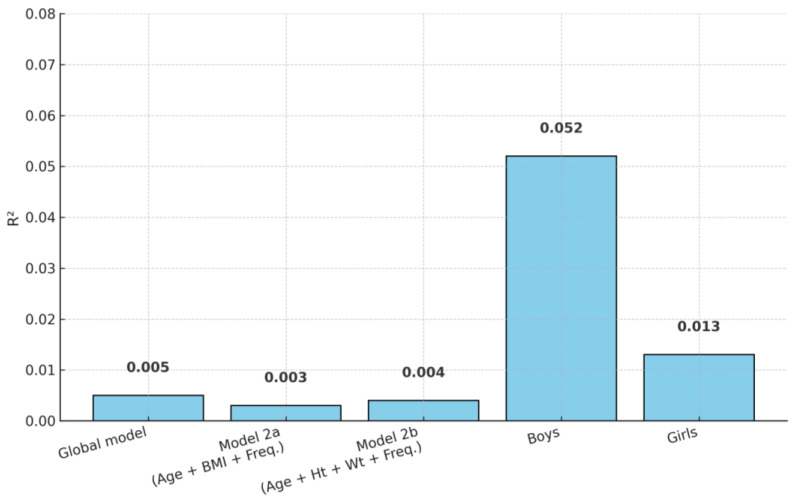
Explained variance (R^2^) in turn performance by Regression Models. Note. None of the models reached statistical significance. All R^2^ values were below 0.06, indicating minimal explanatory power for turn performance.

Because BMI is derived from both weight and height, collinearity issues were observed when all three variables were entered simultaneously (Figure 2). To address this, two parsimonious models were tested: one including Age, BMI, and Practice Frequency, and another including Age, Height, Weight, and Practice Frequency. Neither model reached significance (Model 2a: F (3, 210) = 0.21, *p* = 0.889, R^2^ = 0.003; Model 2b: F (4, 209) = 0.21, *p* = 0.934, R^2^ = 0.004).

When analyses were conducted separately by sex, no significant predictors were identified. For boys, the model accounted for 5.2% of the variance but did not reach statistical significance (F (3, 97) = 1.77, *p* = 0.158, R^2^ = 0.052). For girls, the explained variance was minimal (F (3, 109) = 0.47, *p* = 0.702, R^2^ = 0.013).

## 4. Discussion

The aim of this study was to analyze the execution level of turning on the transverse and horizontal axes in children aged 9 to 11, considering its relationship with physical growth parameters (height, weight, and BMI), sex, and participation in extracurricular physical and sports activities.

The results of the present study show that, within the age range of 9 to 11 years, turn execution level is mostly situated in medium and medium-low ranges, without evidence of a linear progression with age. This pattern partially aligns with the literature on motor development in middle childhood, a stage characterized by the consolidation and refinement of fundamental skills [18,19]. However, the absence of sustained improvement may indicate that, although neuromuscular and coordinative capacities are expanding, turning as a stabilizing skill requires more specific practice to optimize performance [13,14].

In this regard, it was observed that children tend to fail the turn test when required to perform tasks involving impulse adjustment, particularly when such tasks demand preliminary actions such as running, varying the starting position, or deciding how much force to apply. This finding is consistent with other studies that used the same test and reported similar levels of turn competence [23,24]. These situations appear to add an additional demand for motor control and planning that is not always fully developed at this stage, as they involve anticipating trajectory, regulating angular velocity, and adapting posture according to environmental conditions. This combination of biomechanical, perceptual, and cognitive demands may overload the available motor control resources, which helps to explain why performance is more compromised in contexts requiring rapid and precise adjustments [25]. In this line, this transition from fundamental to specific entails an increase in motor complexity, as well as in decision-making and adaptation to changing situations, integrating cognitive, perceptual, and emotional variables. Consequently, mastery of fundamental skills in middle childhood is an essential prerequisite for later success in more specialized physical and sports activities [26].

Our regression analyses showed that neither anthropometric variables (height, weight, BMI) nor overall physical activity frequency significantly predict performance in turns. This is consistent with studies that indicate that, although anthropometric factors may have small or inconsistent associations with motor coordination in children [27,28], they are not sufficient to explain performance in complex motor tasks. Instead, research highlights the importance of specific sports experience in improving coordination, noting that children who participate in structured sports such as gymnastics or athletics perform better in gross motor skills [29,30]. Taking together, these findings support the idea that the execution of the turn depends primarily on specific task practice and neuromotor coordination, rather than on overall body composition or the total volume of sports practice. Therefore, anthropometric factors such as height, weight, and body mass index influence the mechanics and control of turning, while sports practice promotes the automation of movement and motor response [31,32,33].

The literature emphasizes that learning complex stabilizing skills requires varied and specific experiences that stimulate sensory integration and postural control [8,9], aspects that are not necessarily developed in all sport modalities. Likewise, optimal levels of physical and motor fitness enhance children’s ability to correctly perform actions requiring jumping and turning [34]. Most children with low levels of physical and sports practice show a medium or low level of preparation, which results in technical errors and reduced control during motor actions involving turning.

In terms of gender differences, the results show that boys participate in sports more frequently and perform better at turns than girls. These results may reflect differential socialization patterns and sport preferences between boys and girls at these ages, as well as a possible indirect relationship between higher practice frequency and better performance in specific skills, though limited to contexts where such practice involves technical elements like turning [35]. These differences, although small to moderate in effect size, suggest that greater involvement of children in physical activities may contribute to the improvement of stabilizing skills such as turning. Previous studies have documented that systematic and varied practice promotes the automation of motor patterns and enhances overall motor competence [36,37]. Likewise, it has been reported that girls tend to participate in physical and sporting activities less frequently and in contexts less oriented towards the development of specific motor skills [26] which could explain part of the observed difference. However, it is important to note that gender differences do not reflect innate limitations, but rather inequalities in opportunities for practice and sociocultural expectations linked to sport.

Taken together, the findings reinforce the importance of addressing turning as a stabilizing skill that requires intentionally designed training and practice experiences, beyond general physical activity. Furthermore, the data suggests that school and extracurricular interventions should prioritize specific physical and sporting activities where turning plays a major role, such as gymnastics and acrobatics, dance, or martial arts. The variability of contexts may facilitate the transfer of fundamental skills to more complex situations, as highlighted by Newell [38] and Santos et al. [39]. The limited influence of anthropometric variables and general practice frequency underscores the central role of teaching quality and motor content in the development of this skill.

This study has certain limitations that should be considered. First, its cross-sectional design does not allow for the establishment of developmental trajectories or causal relationships between growth parameters, practice habits, and motor performance. Second, the assessment of extracurricular physical and sports activities was based on frequency and self-reported type of activity, without considering the quality or specificity of the practice, which may limit the interpretation of its relationship with turning skills. Furthermore, the assessment was limited to a single motor test, which may not fully reflect the complexity of turning as a stabilizing skill in various contexts.

Another important limitation relates to the type of sampling. Selection based on convenience and availability, although common in school research, restricts the external validity of the study and limits the generalization of findings to broader populations or different contexts. This means that the results should be interpreted with caution and understood primarily as an exploratory approach that provides initial evidence on turning ability in middle childhood.

Finally, the sample was limited to a specific age range and sociocultural context, which restricts the generalization of the results to broader populations.

Future research should adopt longitudinal approaches to better understand the progression of turning skills throughout late childhood and adolescence, considering the combined influence of neuromotor maturation, pubertal growth, and accumulated practice. In addition, it is important to examine how the quality and type of practice affect this skill, comparing sports with high rotational demands, such as gymnastics, dance, or martial arts, with those emphasizing predominantly linear movements, such as athletics or swimming. Such comparisons may clarify the extent to which specific practice experiences enhance postural control and motor adaptation. Furthermore, future studies should investigate the perceptual-cognitive components involved in turning, including anticipatory control, motor planning, and multisensory integration. Combining motor tests with neurocognitive assessments could provide a more comprehensive understanding of the factors that determine successful performance in this stabilizing skill.

## 5. Conclusions

This study showed that, in children aged 9 to 11, the execution level of turns on the transverse and horizontal axes is predominantly situated within medium and medium-low ranges (overall M = 6.8, SD = 2.0), with no linear progression associated with age. Regression analyses confirmed the absence of significant associations with anthropometric variables (height, weight, BMI) or with frequency of sports practice (all *p* > 0.05), suggesting that performance in this motor skill is directly determined not by body composition or the amount of activity, but rather by the quality and specificity of motor experiences. Regarding sex differences, boys outperformed girls at ages 9 (M = 6.5 vs. 5.9, d = 0.35) and 10 (M = 7.7 vs. 6.9, d = 0.41), although these differences diminished by age 11 (M = 6.9 vs. 6.8, ns).

Overall, the findings highlight that turning, as a complex stabilizing skill, requires targeted and contextualized teaching and practice that promote motor anticipation, postural regulation, and coordinative control. Therefore, both school and extracurricular interventions should be designed to provide experiences that incorporate variability, perceptual challenge, and technical specificity, fostering the transfer of fundamental skills to more complex motor situations.

## Figures and Tables

**Table 1:** Scale for assessing the motor skill of turning, according to age [22].

Tasks	Age and Corresponding Difficulty Level					
	5–6 Years	7 Years	8 Years	9 Years	10 Years	11–12 Years
T1. A quarter turn (90°) jump, landing on the same take-off spot.	L	L				
T2. A quarter turn (90°) jump, landing on the same take-off spot with feet together and arms raised.	L	L				
T3. Forward tucked roll starting from a crouching position, finishing seated with legs extended and apart, arms raised.	M	ML	L	L	L	L
T4. A quarter turn (90°) jump from an elevated surface (vaulting box at knee height), landing with feet together.	ML	ML	L	L	L	L
T5. Forward tucked roll starting from a crouching position, finishing seated with legs bent and together, feet flat on the mat and hands on the knees.	MH	ML	L	L	L	L
T6. A quarter turn (90°) jump onto an elevated surface (top of the vaulting box), landing with feet together and arms extended to the sides.	M	ML	ML	L	L	L
T7. Standing inside a 60 cm diameter circle, a half turn (180°) jump, landing inside the circle with feet together and arms raised.	M	M	ML	ML	L	L
T8. Forward roll starting from a standing position with legs straight and together, finishing seated with legs open and extended, arms raised.	MH	M	ML	ML	L	ML
T9. Short run of 8–10 m and, without stopping, a half turn (180°) jump, landing on the take-off spot with arms raised.	H	MH	M	M	ML	ML
T10. Standing inside a 60 cm diameter circle, a three-quarter turn (270°) jump, landing inside the circle with feet together and arms extended to the sides.		H	MH	MH	ML	M
T11. Short run of 8–10 m and, without stopping, a three-quarter turn (270°) jump, landing on the take-off spot with feet together and arms extended to the sides.		H	H	MH	MH	MH
T12. Forward tucked roll starting from a squatting position, finishing in the same squatting position with arms forward, without using the hands or arms to push up.				H	H	H
T13. Backward roll starting from a standing position with legs open, finishing standing with legs open and extended, trunk bent forward and arms forward.				H	H	H
T14. Backward tucked roll starting from a crouching position, finishing in a squatting position with arms forward.				H	H	H
T15. Standing inside a 60 cm diameter circle, a full turn (360°) jump, landing inside the circle.					H	H

Note: Performance levels were coded as L = Low, ML = Medium-Low, M = Medium, MH = Medium-High, and H = High. Each task was scored dichotomously (1 = correct execution; 0 = incorrect), following the standardized test protocol.

**Table 2:** Scores for levels of motor competence in turning by age.

Age	Low	Medium-Low	Medium	Medium–High	High
9 year s	0–4 points	5–6 points	7 points	8–9 points	10–12 points
10 year s	1–6 points	7–8 points		9 points	10–13 points
11 year s	1–5 points	6–7 points	8 points	9 points	10–13 points

**Table 3:** Motor competence level in turning, by age and sex.

Age	Sex	n	x̅ (SD)	L%	ML%	M%	MH%	H%
9 years	Boy	29	6.52 (1.93)	6.9	41.4	13.8	37.9	0.0
	Girl	38	5.87 (1.72)	13.2	52.6	21.1	13.2	0.0
	Total	68		10.5	47.8	17.9	23.8	0.0
10 years	Boy	44	7.73 (2.08)	18.1	63.7	0.0	0.0	18.1
	Girl	36	6.89 (1.90)	30.5	63.8	0.0	2.8	2.8
	Total	80		23.9	63.8	0.0	1.3	11.3
11 years	Boy	28	6.93 (2.26)	35.7	0.0	39.3	17.9	7.1
	Girl	39	6.79 (2.02)	25.7	18.0	43.6	10.3	2.6
	Total	68		29.9	10.5	41.8	13.4	4.5

**x̅**: Mean; SD: Standard deviation; L: Low; ML: Medium-low; M: Medium; MH: Medium-high; H: High.

**Table 4:** Anthropometric data by age and sex.

Age	Sex	n	Height (m)	Weight (kg)	BMI	BMI Z-Score (Who)
9 years	Boy	29	1.38 (0.103)	36.90 (10.97)	18.837 (3.15)	1.07 (1.40)
	Girl	38	136 (0.056)	35.48 (8.10)	19.034 (3.53)	0.93 (1.39)
10 years	Boy	44	1.43 (0.060)	40.59 (9.50)	19.55 (3.67)	0.82 (2.08)
	Girl	36	1.44 (0.072)	41.22 (7.75)	19.67 (3.05)	1.00 (1.30)
11 years	Boy	28	1.49 (0.077)	43.936 (8.96)	19.43 (2.90)	0.52 (1.75)
	Girl	39	1.49 (0.071)	44.82 (10.62)	19.88 (3.95)	0.58 (1.73)

**Table 5:** Gender differences in the frequency of sports practice and the level of turns.

Variable	Boys M (SD)	Girls M (SD)	t (df)	*p*	Cohen’s d
Frequency of physical activity	2.79 (0.97)	2.32 (1.05)	3.42 (212)	<0.001	0.47
Level Turn	7.16 (2.14)	6.51 (1.93)	2.32 (212)	0.021	0.30

## Data Availability

The data presented in this study are available on request from the corresponding author due to ethical and privacy restrictions. The dataset includes information from minors, and the informed consent approved by the institutional ethics committee does not allow public deposition of individual-level data. De-identified data and analysis code can be shared upon reasonable request for bona fide research purposes.
